# Genomic landscape of rat strain and substrain variation

**DOI:** 10.1186/s12864-015-1594-1

**Published:** 2015-05-06

**Authors:** Roel Hermsen, Joep de Ligt, Wim Spee, Francis Blokzijl, Sebastian Schäfer, Eleonora Adami, Sander Boymans, Stephen Flink, Ruben van Boxtel, Robin H van der Weide, Tim Aitman, Norbert Hübner, Marieke Simonis, Boris Tabakoff, Victor Guryev, Edwin Cuppen

**Affiliations:** Hubrecht Institute, KNAW and University Medical Center Utrecht, Uppsalalaan 8, 3584 CT Utrecht, The Netherlands; Max Delbrück Center for Molecular Medicine, Berlin, Germany; Department of Pharmacology, University of Colorado School of Medicine, 12800 E. 19th Ave., Aurora, CO USA; Physiological Genomic and Medicine Group, MRC Clinical Sciences Centre, London, UK; European Research Institute for the Biology of Ageing, University of Groningen, University Medical Centre Groningen, Antonius Deusinglaan 1, 9713 AD Groningen, The Netherlands

**Keywords:** Rat, Inbred strain, Substrain, Genomic variation, RGSC5.0, rn5, rnor5.0

## Abstract

**Background:**

Since the completion of the rat reference genome in 2003, whole-genome sequencing data from more than 40 rat strains have become available. These data represent the broad range of strains that are used in rat research including commonly used substrains. Currently, this wealth of information cannot be used to its full extent, because the variety of different variant calling algorithms employed by different groups impairs comparison between strains. In addition, all rat whole genome sequencing studies to date used an outdated reference genome for analysis (RGSC3.4 released in 2004).

**Results:**

Here we present a comprehensive, multi-sample and uniformly called set of genetic variants in 40 rat strains, including 19 substrains. We reanalyzed all primary data using a recent version of the rat reference assembly (RGSC5.0 released in 2012) and identified over 12 million genomic variants (SNVs, indels and structural variants) among the 40 strains. 28,318 SNVs are specific to individual substrains, which may be explained by introgression from other unsequenced strains and ongoing evolution by genetic drift. Substrain SNVs may have a larger predicted functional impact compared to older shared SNVs.

**Conclusions:**

In summary we present a comprehensive catalog of uniformly analyzed genetic variants among 40 widely used rat inbred strains based on the RGSC5.0 assembly. This represents a valuable resource, which will facilitate rat functional genomic research. In line with previous observations, our genome-wide analyses do not show evidence for contribution of multiple ancestral founder rat subspecies to the currently used rat inbred strains, as is the case for mouse. In addition, we find that the degree of substrain variation is highly variable between strains, which is of importance for the correct interpretation of experimental data from different labs.

**Electronic supplementary material:**

The online version of this article (doi:10.1186/s12864-015-1594-1) contains supplementary material, which is available to authorized users.

## Background

The rat is an important model organism for studying human disease biology [1]. In the past century, a great variety of strains and substrains have been bred that differ in susceptibility to complex diseases like hypertension, diabetes, autoimmunity, cancer and addiction disorders. Due to practical limitations, studies on disease phenotypes are often conducted in varying substrains by different research groups. For example, SHR/NCrl and SHR/NHsd are used for studying cardiovascular phenotypes in the United States [2] and Europe [3], respectively. The effect on the interpretability and extrapolation of the obtained results by using different substrains remains unclear. Several studies based on DNA SNP marker panels showed that genetic variation between substrains is present [4-6]. However, the magnitude of this difference can only be properly interpreted when assessed on a genome-wide scale as variation is not necessarily randomly distributed throughout the genome. Here, we systematically (re)analyzed whole genome sequence (WGS) data of 40 rat strains and substrains resulting in a comprehensive inventory of strain and substrain-specific variants.

With the emergence of next-generation sequencing (NGS) techniques, whole genome sequencing of many rat strains and substrains were performed [7-12], with the primary goal to provide insight in the genetic factors underlying phenotypic traits in these strains. After the availability of the first rat reference genome assembly in 2003 [13], the first variation catalog of a non-reference inbred strain, the spontaneously hypertensive rat (SHR), was published in 2010 [7]. This data was later combined with the BN-*Lx* genome sequence and extended with RNA sequencing data, resulting in a comprehensive catalog of genetic variation and associated quantitative and qualitative transcription phenotypes, in the HXB/BXH recombinant inbred (RI) panel [8]. This panel is a valuable tool for dissection of the complex genetic basis of cardiovascular, behavioral, and developmental disorders. In addition, the eight founders of the rat heterogeneous stock (NIH-HS) were recently sequenced [9]. In this study, the genome sequence of the founder strains were used to impute the genomes of the 1407 SNP-genotyped heterogeneous stock rats that were also extensively phenotyped. This work resulted in the identification of 355 high-resolution quantitative trait loci (QTLs) for 122 phenotypes. More rat whole genome sequence data became available by publication of the variation catalog and strain specific sequences of the Dark Agouti (DA) and Fischer (F344) rat, which carry unique dichotomous phenotypes, such as rheumatoid arthritis and several cancer types [10]. Finally, a large community-driven effort in rat genome sequencing yielded variation catalogs of 25 inbred strains and substrains [11]. Analysis of this data identified strain-specific selective sweeps and gene clusters that implied genes involved in the development of cardiovascular disease in rat.

One important factor that determines the success of cataloging genomic variation is the quality of the used reference genome. Since its initial publication in 2003, the rat reference genome has undergone major improvements and was recently further improved using a range of NGS-based methods [14]. This has resulted in version 5.0 of the rat reference assembly in 2012 [15]. Although the v5.0 assembly shows great overall improvement at both nucleotide and the structural level, it has not yet been used as a reference for the analysis of the aforementioned rat genomes. Instead, these studies all used the v3.4 assembly, which is publicly available since 2004 [13] and contains many gaps, assembly inconsistencies and nucleotide and indel errors (due to the relatively low coverage and typical errors associated with capillary dideoxy sequencing).

Finally, bioinformatic analysis of whole genome sequencing data, including mapping and variant calling, has matured rapidly over the past years. However, as a result of these ongoing developments, a broad range of bioinformatic tools and settings were used for the analysis of currently published rat genomes. Direct comparison of different strains therefore becomes challenging, especially because many old tools did not call reference positions. Taken together, a comprehensive overview and systematic comparison of laboratory rat genomic variation is currently lacking. Such a resource would be useful for a broad range of rat researchers, as it allows proper selection of experimental and control rat strains and interpretation of potential substrain effects in published experiments.

## Results

### Genetic variation among strains

We gathered the genomes of 37 rat strains that were sequenced previously [7-12] (Table 1) and analyzed them together with newly derived sequences from the BN-*Lx*/CubPrin, SHR/OlaIpcvPrin and SHR/NCrlPrin rat strains (Additional file 1). We aligned reads of all 40 strains to the RGSC5.0 assembly (BN/NHsdMcwi; [13]). After applying strict criteria (see Methods) and using multi-sample variant calling we identified in total 9,183,702 SNVs, 3,001,935 indels and 63,664 structural variants compared to the reference assembly.

**Table 1:** **Sequence variation in 40 + 1 rat strains**

**Rat strain**	**Publication**	**PMID**	**Sequencing platform**	**Number of SNVs**	**Number of indels**	**Number of structural variants**
ACI/EurMcwi	Atanur et al.	23890820	Illumina HiSeq2X00	3,539,775	1,651,251	7,259
ACI/N	Baud et al.	23708188	SOLiD 4 and 5500	3,125,523	1,382,793	19,541
BBDP/Wor	Atanur et al.	23890820	Illumina HiSeq2X00	3,279,444	1,526,223	3,678
BN/SsN	Baud et al.	23708188	SOLiD 4 and 5500	59,402	660,918	14,126
BN-*Lx*/Cub	Simonis et al.; Atanur et al.	22541052; 23890820	SOLiD 2,3 and 4	102,359	627,056	13,391
BN-*Lx*/CubPrin	Hermsen et al.	na	Illumina HiSeq2000	140,376	420,433	13,410
BUF/N	Baud et al.	23708188	SOLiD 4 and 5500	2,848,992	1,302,710	18,481
DA/BklArbNsi	Guo et al.	23695301	Illumina HiSeq2000	3,368,008	1,567,160	4,184
F334/N	Baud et al.	23708188	SOLiD 4 and 5500	2,947,509	1,342,709	20,881
F344/NCrl	Atanur et al.	23890820	Illumina HiSeq2X00	3,369,205	1,579,418	3,492
F344/NHsd	Guo et al.	23695301	Illumina HiSeq2000	3,367,166	1,573,573	3,950
FHH/EurMcwi	Atanur et al.	23890820	Illumina HiSeq2X00	3,389,304	1,592,915	3,011
FHL/EurMcwi	Atanur et al.	23890820	Illumina HiSeq2X00	3,361,824	1,586,543	8,504
GK/Ox	Atanur et al.	23890820	Illumina HiSeq2X00	3,549,952	1,575,619	4,241
LE/Stm (Illumina)	Atanur et al.	23890820	Illumina HiSeq2X00	3,412,610	1,578,099	2,598
LE/Stm (SOLiD)	Baud et al.	23708188	SOLiD 4 and 5500	2,949,814	1,359,947	21,038
LEW/Crl	Atanur et al.	23890820	Illumina HiSeq2X00	2,884,477	1,409,659	3,642
LEW/NCrl	Atanur et al.	23890820	Illumina HiSeq2X00	2,884,763	1,402,459	3,996
LH/MavRrrc	Atanur et al.; Ma et al.	23890820; 24628878	Illumina HiSeq2X00	3,369,852	1,584,236	2,891
LL/MavRrrc	Atanur et al.; Ma et al.	23890820; 24628878	Illumina HiSeq2X00	3,329,343	1,565,343	3,070
LN/MavRrrc	Atanur et al.; Ma et al.	23890820; 24628878	Illumina HiSeq2X00	3,319,381	1,562,698	2,952
M520/N	Baud et al.	23708188	SOLiD 4 and 5500	2,896,825	1,321,431	19,308
MHS/Gib	Atanur et al.	23890820	Illumina HiSeq2X00	3,183,312	1,513,330	2,917
MNS/Gib	Atanur et al.	23890820	Illumina HiSeq2X00	3,168,796	1,538,413	3,105
MR/N	Baud et al.	23708188	SOLiD 4 and 5500	2,878,806	1,350,411	18,001
SBH/Ygl	Atanur et al.	23890820	Illumina HiSeq2X00	3,393,610	1,617,252	14,787
SBN/Ygl	Atanur et al.	23890820	Illumina HiSeq2X00	3,300,171	1,592,247	15,216
SHR/NCrlPrin	Hermsen et al.	na	Illumina HiSeq2000	3,736,435	1,694,012	14,179
SHR/NHsd	Atanur et al.	23890820	Illumina HiSeq2X00	3,756,155	1,705,126	3,950
SHR/OlaIpcv	Simonis et al.; Atanur et al.	22541052; 23890820	Illumina Genome Analyser 2	3,747,579	1,706,963	4,066
SHR/OlaIpcvPrin	Hermsen et al.	na	Illumina HiSeq2000	3,709,362	1,689,758	14,069
SHRSP/Gla	Atanur et al.	23890820	Illumina HiSeq2X00	3,700,495	1,723,961	2,301
SR/Jr	Atanur et al.	23890820	Illumina HiSeq2X00	3,353,579	1,568,778	3,699
SS/Jr	Atanur et al.	23890820	Illumina HiSeq2X00	3,311,117	1,553,050	3,685
SS/JrHsdMcwi	Atanur et al.	23890820	Illumina HiSeq2X00	3,310,209	1,595,799	7,938
SUO_F344	Hermsen et al.	na	Illumina HiSeq2000	3,349,024	1,549,272	11,864
WAG/Rij	Atanur et al.	23890820	Illumina HiSeq2X00	3,092,505	1,485,673	3,650
WKY/Gla	Atanur et al.	23890820	Illumina HiSeq2X00	3,777,400	1,725,868	3,292
WKY/N	Baud et al.	23708188	SOLiD 4 and 5500	3,213,913	1,419,460	21,832
WKY/NCrl	Atanur et al.	23890820	Illumina HiSeq2X00	3,502,459	1,700,646	3,630
WKY/NHsd	Atanur et al.	23890820	Illumina HiSeq2X00	3,682,736	1,665,949	4,691
WN/N	Baud et al.	23708188	SOLiD 4 and 5500	2,899,096	1,323,116	18,995

Sequence information from 40 known strains was used. The unknown SUO_F344 strain was also included in the analysis. In addition LE/Stm was sequenced with two separate sequencing platforms: Illumina and SOLiD; these two datasets were treated as separate samples in the analysis. Therefore in total this table contains variant information of 42 samples from 40 + 1 rat strains.

To assess the sensitivity and specificity of our calls we made use of finished capillary sequencing data from 13 BAC clones from the LE/Stm strain, which was also sequenced by two different NGS approaches. We evaluated 2,132,438 nucleotides and found in total 2,468 SNVs that were detected by capillary sequencing and NGS techniques. 141 SNVs were missed by whole-genome sequencing; resulting in an estimate of 524,677 (5.4%) missed SNVs genome-wide. 14 SNVs identified by whole-genome sequencing were not found in the BACs; resulting in an estimate of 55,817 (0.6%) false positive SNV calls genome-wide. For indels the false positive and negative call rates are higher (FP:15,7% FN:27,3%) due to known detection difficulties of current calling algorithms. Although the 40 strains were sequenced on two different NGS platforms (SOLiD and Illumina), false positive and negative call rates based on the LE data (sequenced on both platforms) were similar (Additional file 2).

#### Small genomic variation: SNVs and indels

We identified single nucleotide variants and small insertions and deletions (indels) with the Genome Analysis Toolkit (GATK) HaplotypeCaller [16]. All together we identified 9.2 M SNVs of which 97.5% were homozygous and 2.5% were heterozygous. This small percentage of heterozygous variants can be attributed to incomplete fixation of the inbred strain, genomic duplications followed by diversification, and technical errors in the sequencing or data analysis. These variants were filtered out in a separate file (see Availability of Supporting Data) and were not taken into account in further downstream analyses.

**Figure 1:**
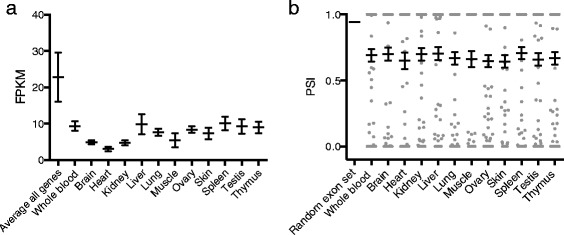
‘Repression’ of genes and exons containing high impact SNVs. **(a)** Genome-wide average FPKM ± SEM across all tissues compared to the average FPKM of genes containing high impact SNVs for 12 tissues. Genes containing high impact SNVs are significantly lower expressed (Non-parametric ANOVA; p < 0.0001). **(b)** The average Percentage Spliced In (PSI) ± SEM across the transcriptome was compared to the average PSI of exons containing high impact SNVs for 12 tissues. Exons containing high impact SNVs are significantly more spliced out/not used (Non-parametric ANOVA; P < 0.0001).

To understand the functional consequences of the SNVs we annotated these variants using SnpEff (Table 2) [17]. Predictions on the functional consequences of a variant are typically overestimated due to for instance their presence in pseudogenes or non-constitutive exons [18]. Here we set out to systematically interrogate the extent of this overestimation by a detailed dissection of 601 SNVs which are annotated to have a deleterious effect (marked as causing “HIGH” impact by SnpEff) on gene function including stop-gain mutations and alterations of splice sites (Table 2). First we tested the hypothesis that neighboring variants could possibly restore the open reading frame by investigating the high impact SNV vicinity. We found for 60 SNVs (10%) a neighboring SNV or indel that restored the open reading frame (Additional file 3). From the remaining 541 high impact SNVs we determined the expression in twelve BN-*Lx*/Cub tissues for the genes in which the variants are located (Figure 1). We then compared this to the expression of all genes and found that the highly impacted genes are expressed at significantly lower levels (non-parametric ANOVA; p < 0.0001). In addition, for the expressed genes, we analyzed the usage of individual exons by means of the ‘Percentage Spliced In’ (PSI) index per exon. Interestingly, we found that the exons containing high impact SNVs tend to be less used and more often spliced out than expected (non-parametric ANOVA; P < 0.0001). Thus, we conclude that most high impact SNVs will actually only have a limited biological relevance, in part by neutralization by neighboring variants or by being ‘repressed’ in expression at the gene and exon levels.

**Table 2:** Prediction of the functional consequences of the SNVs

**Type**	**Impact**	**Count**	**Fraction**	**Sum**
Stop gained	High	285	0.0%	696
Splice site donor		209	0.0%	
Splice site acceptor		158	0.0%	
Start lost		26	0.0%	
Stop lost		18	0.0%	
Non synonymous coding	Moderate	26,239	0.3%	26,239
Synonymous coding	Low	42,182	0.4%	42,947
Start gained		725	0.0%	
Synonymous stop		35	0.0%	
Non synonymous start		5	0.0%	
Intergenic	Modifier	6,509,332	62.2%	10,394,771
Intron		2,991,180	28.6%	
Downstream		430,875	4.1%	
Upstream		427,613	4.1%	
UTR 3 Prime		27,145	0.3%	
UTR 5 Prime		4,357	0.0%	
Exon		4,269	0.0%	
**Total effects**		**10,464,653**		**10,464,653**

#### Cross-species comparison of genome variation

**Figure 2:**
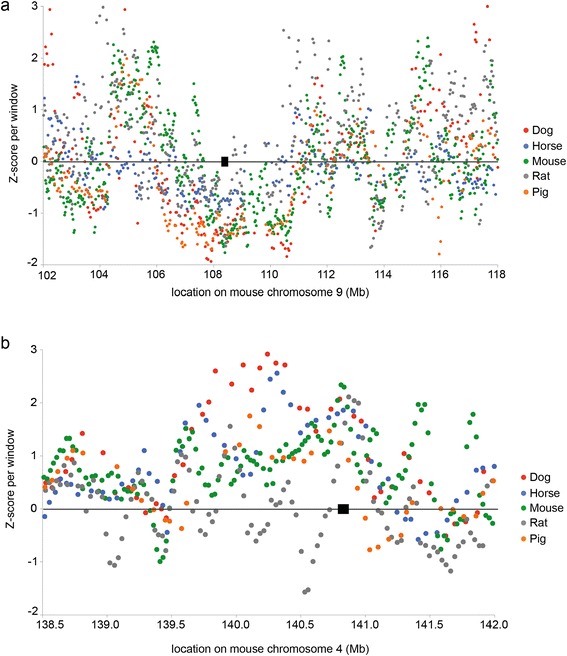
Cross-species comparison of SNV densities. **(a)** An example of a locus (black rectangle) on mouse chromosome 9 with the lowest SNV density in five domesticated species. **(b)** An example of a locus (black rectangle) on mouse chromosome 4 with the highest SNV density in five domesticated species.

To get an impression of the nucleotide diversity among laboratory rat strains in relation to other domesticated animals, we compared the SNV density between five different domesticated species. We extracted all autosomal genomic regions that are one-to-one comparable (syntenic) with the rat genome from dog, horse, pig and mouse. Next, we determined the amount of species-specific SNVs in each 100 kilobase syntenic window to identify regions that contain high and low nucleotide diversity in each species. We extracted the regions with highest and lowest amount of SNVs that are shared among all five species. In total, the cumulative regions with a low SNV density contain 28 genes at 4 genomic loci (Figure 2a). When we functionally annotate these genes using the PANTHER Classification System [19], we find enrichment (p < 0.05) for genes involved in catabolic processes (Additional file 4). This might reflect the evolutionary constraint on diet, exerted in these five species by domestication [20]. For the regions that exhibit high SNV density in all five species we in total find 51 genes at 6 genomic loci (Figure 2b). Functional annotation with PANTHER shows an enrichment (p < 0.001) for olfactory and hemoglobin genes, which are known to rapidly evolve and are highly variable in several species [21,22].

Another way to look at loci under selective pressure is by studying the non-synonymous to synonymous substitution rate per gene (K_a_/K_s_ ratio). Genes that are potentially under positive selection have a non-synonymous to synonymous ratio of >1.0 [23]. We identified all protein coding genes (n = 22,941) that contain 6 or more SNVs in the protein-coding region (n = 3,006) and extracted the genes that have a non-synonymous to synonymous ratio of >1.0 (n = 909). PANTHER functional annotation of these 909 genes using the 3,006 genes as background shows that this set is enriched for genes related to the immune and olfactory system (p < 0.05; Additional file 5). This data confirms the results of the interspecies SNV density analysis and shows that within rat strains these types of genes are indeed highly polymorphic [21].

#### ‘Population’ structure

**Figure 3:**
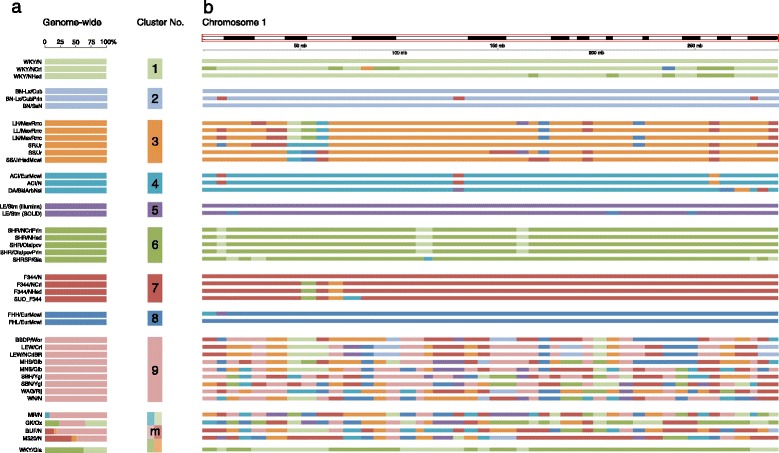
‘Population’ structure of 40 + 1 rat strains. **(a)** Per strain, the contribution from the 9 different clusters is plotted as percentage of the genome. Each cluster is represented by a separate color. The cluster designated with a ‘m’ represents the strains that have membership from multiple clusters. **(b)** Per strain, the genomic distribution along rat chromosome 1 is plotted as an example. The colors match the cluster colors from **(a)**.

To get an impression of the ‘population’ structure of these 40 strains, we used the SNV genotype information per locus in a Bayesian approach to define clusters without any other prior knowledge. In addition, to demonstrate the power of this approach to accurately define clusters, we included genotypes from WGS data from a Strain of Unknown Origin (SUO). We hypothesized that we would be able to designate the strain of origin based on the genotypes of a broad representation of rat strains in this data set. We performed this analysis using fastStructure, which is an algorithm for inferring population structure from large SNP genotype data [24]. fastStructure identifies the number of populations (clusters or ‘K’) needed to explain the structure in the data in which individual samples can have membership in multiple clusters. When we analyze the genotypes of all 40 + 1 rat strains we find that we can differentiate nine distinct clusters (Figure 3a). Five strains have membership in multiple clusters, which may reflect shared ancestry or interbreeding before or during inbred strain derivation, whereas the other strains only consist of one cluster. In general most clusters resemble the previously published classification based on a rooted phylogenetic tree [11]. In addition this method allows identification of similarity between clusters that have been separated in a phylogenetic tree analysis. For example, the GK/Ox strain, which is a Wistar derived strain originating from Japan, also shows contribution of the cluster which contains the Wistar derived strains from Europe and the United States [11]. We also find that the included SUO strain clearly shows a full match in the Fischer (F344) cluster and we therefore conclude that the SUO is a substrain of the F344 strain (SUO_F344). Besides the ancestral clustering of strains, we also studied the subchromosomal pattern of similarity and divergence. We determined for each bin of 20,000 SNVs to which cluster it was most similar (Figure 3b and Additional file 6). Based on this analysis we observed that the overall clustering based on the genomes as a whole, matches the clusters found in the genomic cluster distribution using the 40 + 1 strains and is concordant with previous work [11]. We find that substrains (e.g. the SHR substrains) have a comparable genomic cluster structure, indicating recent divergence. Of note, the relatively large window size of 20,000 SNVs may cause overrepresentation of differential loci between substrains that are known to be very similar (e.g. the Lyon strains [12]). Nevertheless, we find five rat strains that showed contribution from multiple clusters in the fastStructure analysis (group ‘m’) of which one (WKY/Gla) shows a genomic distribution of the clusters #1 (WKY) and #6 (SHR), which is in line with its known breeding origin [11,25]. In addition, cluster 9 (with e.g. the LEW substrains) shows a confetti-like signature, while the fastStructure analysis does not categorize them as multi-cluster strains. In conclusion, we see shared haplotypes between strains in different clusters, indicating common ancestry and/or cross-breeding during inbred strain derivation. Nevertheless, the variation uniqueness per cluster is very high.

#### Large genomic variation: structural variants

Structural variants were determined using two independent methods. I) We interrogated the orientation of the mapped read-pairs (RP) compared to the reference genome to detect deletions, tandem duplications and inversions by applying the DELLY [26] algorithm in all samples simultaneously. II) CNVnator [27] was used to identify relative changes in read-depth (RD) thereby detecting losses and gains of genomic segements. Given the algorithmic difficulties in detecting structural variants we took a strict cutoff to minimize false positive calls (see Methods). In total, we identified 34,433 deletions, 585 tandem duplications and 26,899 inversions based on the read-pair method together with 1,747 copy number variable sites based on the read-depth method. All together this resulted in 63,664 SVs in the 40 strains.

### Substrain variability

To identify the genomic variants that differ between substrains we used the seven strains of which data for at least two substrains was available: ACI, BN, F344, LEW, SHR, SS and WKY (Table 3). We did not include WKY/Gla because this substrain is known to have diverged significantly from the other WKY substrains [25] which is also evident from our genomic comparisons. For each of the seven groups we identified all positions that were variable between the substrains. We found that the degree of substrain variation was highly variable between strains (1,046-10,250 per strain) (Table 3), which may reflect the time after separation of the substrain colonies. For comparative functional analyses of substrain variation (detailed below) we used all other SNVs (8,863,815), excluding variants that were shared by all 40 strains, as a comparison group.

**Table 3:** Strains and substrains included in the substrain variability analysis

**Strain**	**Substrains**	**Substrain SNVs**
ACI	ACI/N	3,432
	ACI/EurMcwi	
BN	BN-*Lx*/Cub	2,291
	BN-*Lx*/CubPrin	
	BN/SsN	
F344	F334/N	5,854
	F344/NHsd	
	F344/NCrl	
	SUO_F344	
LEW	LEW/Crl	1,046
	LEW/NCrlBR	
SHR	SHR/OlaIpcv	2,950
	SHR/NCrlPrin	
	SHR/NHsd	
	SHR/OlaIpcvPrin	
SS	SS/Jr	2,495
	SS/JrHsdMcwi	
WKY	WKY/N	10,250
	WKY/NCrl	
	WKY/NHsd	
Total		28,318

**Figure 4:**
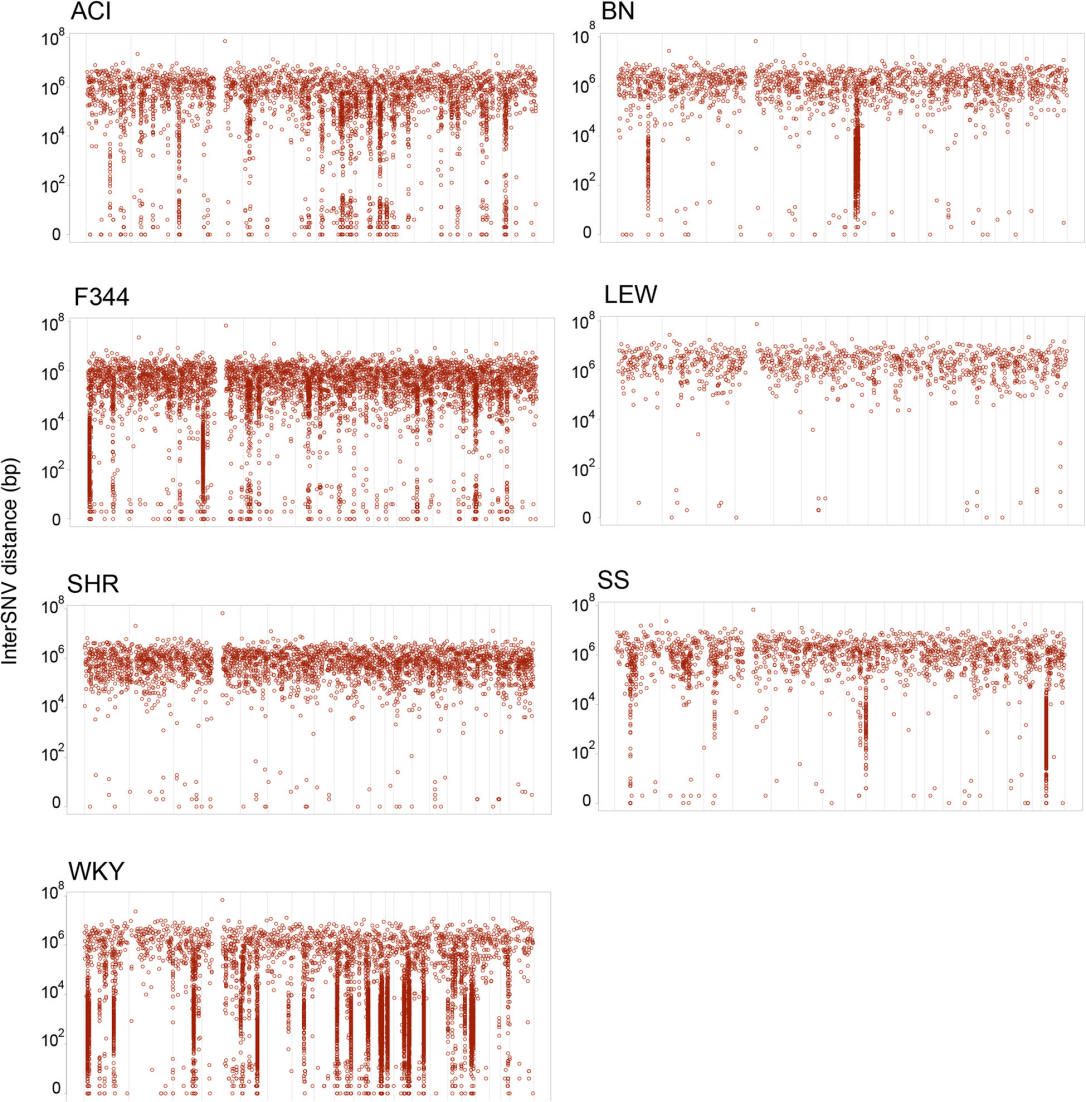
Genomic distribution of substrain variants per strain. For each strain the distance between two consecutive SNVs (y-axis) is plotted along the genomic position (x-axis). The windows on the x-axis represent the different chromosomes. Loci with a high density of substrain SNVs can be observed as clusters that drop down from the average genome-wide pattern.

To get an impression of the genomic distribution of the substrain SNVs we plotted the genomic distance between two consecutive substrain SNVs (Figure 4). For two groups (LEW and SHR) we found an even distribution of the SNVs through the genome, while in the other five groups we also observe clustering of SNVs. This effect is limited to a few loci for BN, but is more widespread for WKY. One explanation for the clustering of these SNVs can be introgression from a rat strain that is not included in the current analysis. For instance we observe a cluster of SNVs in the BN group on chromosome 8. For the BN-*Lx* substrains that are in this group, this region is known to contain the Lx locus from the polydactylous PD/Cub strain [28]. Since whole genome sequencing data of the PD/Cub strain is not available we observe the congenic Lx segment as an introgressed cluster of substrain-specific SNVs in our analysis. Although this analysis is able to identify introgressed loci from other sequenced strains, we cannot exclude that we miss introgression from closely related strains with limited SNV diversity.

**Figure 5:**
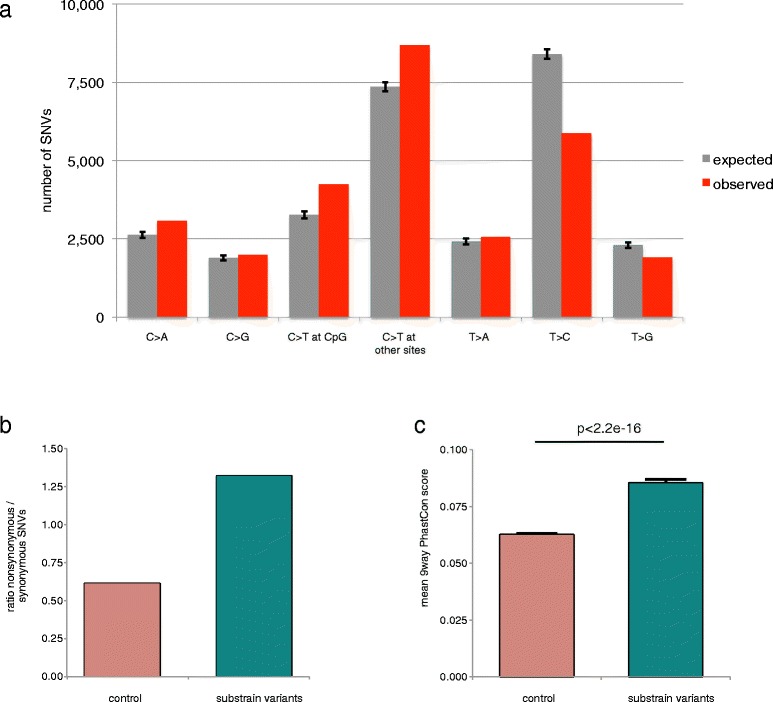
Substrain variant characteristics. **(a)** Bar plots showing the contribution of each nucleotide change for all substrain variants (observed) versus the control variants (expected). Error bars represent the 95% confidence interval. **(b)** Bar plot showing the K_a_/K_s_ ratio ratio of the substrain variants versus the control variants. **(c)** Bar plot showing the average phastCons score for each substrain variant compared to the control variants. Substrain variants affect nucleotides with a significantly higher phastCons score (Student’s *t*-test; p < 2.2e-16). Error bars represent the SEM.

Besides introgression, the occurrence of de novo mutations (genetic drift) appears the main driver of substrain variation [29]. To understand the process of newly arising variants we analyzed the different types of nucleotide changes that occurred. The control set of 8,863,815 SNVs was used to estimate the expected amount of substitutions per category. The observed amount of nucleotide changes of the 28,318 substrain SNVs was then compared to this expected pattern. We find an enrichment of C to T substitutions in general, which is most pronounced at CpG dinucleotides (Figure 5a). This may reflect an elevated rate of spontaneous/oxidative deamination of 5-methyl-cytosines, which is associated with oxidative DNA damage in animal genomes [30]. In addition, we find a significant (p < 0.05) depletion of T to C changes (expected:8399 observed:5866), which are typically the result of alkylating mechanisms [31,32].

In summary, we find supportive evidence that suggests the occurrence of substrain variants by endogenous reactive oxygen species (ROS); a common source of oxidative DNA damage [33]. Based on the mutational spectrum, non-negative matrix factorization (NMF) can be used to identify more detailed underlying mutational signatures. However, when we carry out such analyses we do not find a significant difference in mutational signature between substrain and control SNVs, suggesting that substrain variation results from common mutational processes and thus represents ongoing evolutionary processes.

Next, we investigated the functional consequences of the 28,318 substrain variants by analyzing the nonsynonymous to synonymous ratio, which we previously used as a measure of selective pressure. Interestingly, we find relatively more nonsynonymous SNVs in the substrain variants compared to the control set, indicating that the substrain SNVs more often affect protein sequence (Figure 5b). To substantiate this finding and to get a gene annotation-independent measure of the functional impact of the substrain variants, we also retrieved the phastCons scores [34] per variant. This score (between 0 and 1) is calculated for each nucleotide in the genome as a measure for evolutionarily constraint and was derived by comparing the rat genome to 8 other species: mouse, dog, cow, opossum, chicken, frog, zebrafish and human. In line with the previous results we find a significantly higher phastCons score of the substrain-affected nucleotides compared to the control set (p < 0.0001; Figure 5c). These two lines of evidence suggest that evolutionary pressure has not (yet) selected against these possibly damaging variants, confirming the relatively young age of the substrain variants. On the other hand, substrain-specific variants may have a relatively large effect on protein function and thus on associated biology and it is therefore extremely important to know this category of variation when comparing experimental results obtained with different rat substrains in different labs.

## Discussion

Although RGSC5.0 was already released in 2012, all whole-genome sequencing studies to date are based on the much older RGSC3.4 assembly. Here, we merge publicly available whole genome sequence data of 40 widely used rat inbred strains and substrains into a comprehensive integrated variant inventory. This resource allows researchers to functionally annotate their data on the more recent RGSC5.0.

Integrated analysis of a large number of strains increases effective genomic coverage and thus improves on variant calling sensitivity. The multi-sample variant calling approach used here, makes optimally use of this [7-12], resulting in a more accurate and more complete set of called variants, especially in strains with lower coverage at a given position. The resulting resource is useful for a broad range of researchers who use rats for studying genetic traits and can easily be exploited. For example, this inventory can be used for choosing strains and substrains for specific experiment or as controls, when knowing their genetic differences in a locus of interest. Another way to use this resource is by coupling it to Quantitative Trait Locus (QTL) data, which is available for many of these strains for a broad range of complex traits [8-10,12,35-39]. This allows for filtering for shared and unique variants between strains with and without the trait to narrow in on potential causal variants. Finally, the resource can be used for strain of origin designation when WGS or genotyping data is available, as exemplified by the SUO_F344 WGS data included in this study.

We showed that the biological relevance of most SNVs that are annotated to have a deleterious effect is limited. In part, this effect can be attributed to the low expression level of the gene or to skipping of exons in which a high impact variant is found. Furthermore, a small part of the automatically predicted deleterious variants appeared false positives caused by the lack of taking neighboring variants into account in the effect prediction. Addressing this effect requires adaptions of the current effect prediction calling algorithms.

When we investigate the population structure of the 40 rat strains, we find a distinction between nine separated clusters, which recapitulates the previously published origin of some of these strains [11]. We see that the genomic variant distribution in more than 65% of the strains (27 out of 40) has a clearly distinct pattern between clusters. In addition, all strains in cluster 9 show a confetti-like genomic distribution of multiple clusters, possibly reflecting their heterogeneous, yet shared, origin. Similar to data from mice [40] we observe introgression of shared haplotypes between strains, suggesting intercrosses in rat strain selection processes. Using SNP marker information in rat, it was already shown that this effect was present [5,6] and here we confirm this observation on a genome-wide scale.

Furthermore, we identified substrain variation in seven rat strains and find that the degree of variation is highly variable between strains. The strain with the highest degree of substrain variation is WKY and part of this variation can be explained by their distribution to different geographical locations before complete inbreeding [25]. When we further investigate the different aspects of substrain variants we can explain part of their origin by introgression and part by ongoing evolution through genetic drift. In general the characteristics of substrain variants matches with their recent origin. Firstly the impact of the substrain variants is relatively high: Substrain variants more often affect protein sequence and nucleotides with high phasCons score. Secondly the substrain variants may show suggestive evidence for endogenous ROS DNA damage, a process that continuously challenges the integrity of DNA [33].

## Conclusion

In summary, we present a comprehensive inventory of uniformly called genomic variants mapped on the RGSC5.0 reference assembly for a range of commonly used inbred rat strains. This resource is valuable for a broad range of researchers that use rats in biomedical and complex genetics research and may facilitate further research on rat functional genomics and interspecies comparison. The knowledge on substrain variation may assist experimental design and improve on the outcome and reproducibility of experimental results between institutes and thus improve the overall quality of biomedical animal research.

All data described in this study is publicly available in the Variant Call Format (VCF) and accessible via the Rat Genome Database (http://rgd.mcw.edu/) for browsing or direct FTP downloading [41]. In addition data from the four newly sequenced strains is also available via PhenoGen Informatics (http://phenogen.ucdenver.edu) [42].

## Methods

### Animals

All experiments were approved by the Animal Care Committee of the Royal Dutch Academy of Sciences according to the Dutch legal ethical guidelines. Experiments were designed to minimize the number of required animals and their suffering. Animals were housed under standard conditions in groups of two to three per cage per sex under controlled experimental conditions (12-hour light/dark cycle, 21 ± 1°C, 60% relative humidity, food and water ad libitum). Health status was monitored weekly.

### Genome and transcriptome sequencing

We performed whole genome sequencing on the rat strains: BN-*Lx*/CubPrin, SHR/OlaIpcvPrin, SHR/NCrlPrin, and SUO_F344. Tissues were obtained from animals of the stock maintained by Dr. Morton Printz, Department of Pharmacology, University of California San Diego. Genomic DNA was extracted from 25 mg of homogenized cortical tissue using the DNeasy Blood and Tissue kit (#69504, Qiagen). One microgram of genomic DNA was used as input in the Illumina TruSeq DNA Kit (#PE-940-2001, Illumina) following the manufacturer’s instructions. The libraries were sequenced using 100 cycles paired-end reads on an Illumina HiSeq2000 following the manufacturer’s instructions.

We performed RNA sequencing on a male BN-*Lx*/Cub of snap-frozen and powdered whole tissues. Total RNA from heart, muscle and skin was isolated was firstly isolated using the TRIzol® reagent (#15596-026, Invitrogen, Life Technologies). After this total RNA was (re)isolated using the Promega Maxwell® 16 MDx Research System (#AS3000, Promega) with the Maxwell® 16 LEV simplyRNA Blood Kit (#AS1310, Promega) for brain, heart, kidney, liver, lung, muscle, ovary, skin, spleen, testis, thymus and whole blood. One microgram of isolated total RNA was used as input for sample prep using TruSeq Stranded Total RNA Kit with Ribo Zero Human/Mouse/Rat (#RS-122-2203, Illumina) following the manufacturer’s instructions. The libraries were sequenced 101 cycles paired-end in rapid run modus on an Illumina HiSeq2500 following standard manufacturer’s instructions.

### Mapping, variant calling and annotation

For the whole genome sequencing data the 32 strains that were sequenced on Illumina platforms were mapped with BWA mem –M 0.7.5a [43]. The 10 strains that were sequenced on SOLiD platform were mapped with BWA 0.5.9 aln -c -l 25 -k 2 -n 10 (the latest version to support color space). Picard MarkDuplicates version 1.89 was used to mark all the duplicate reads per rat strain. SNV and indel calling was done following the GATK HaplotypeCaller v2.8-1-g932cd3a best practices from the Broad Institute [16]. SNVs and indels were annotated using SnpEff version 3.3 h [17]. Structural variant calling was done using DELLY version 0.3.3 with -q 20 [26] and CNVnator version 0.2.7 with a bin size of 1,000 bp [27].

### RNA sequencing downstream analysis

For the RNA sequencing of BN-*Lx*/Cub tissues, reads were mapped to the genome first to detect and remove sequences with multiple alignments. The remaining sequences were then aligned with TopHat 1.4.1 [44] against the RGSC5.0 reference genome and transcriptome based on Ensembl gene annotations [45]. To align reads across both novel and known splice junctions, we also allowed the discovery of unknown splice junctions. We then counted uniquely aligning reads that could be assigned unambiguously to one gene. This count data was then normalized for gene length and library size to obtain genome-wide FPKM values.

‘Percent Spliced In’ (PSI) values were generated by counting reads either mapping into (inclusion read) or jumping over (exclusion read) a given exon. After length normalization, the ratio between inclusion reads was divided by the sum of inclusion and exclusion reads to obtain the PSI score for each exon. As a control, a set of 16,000 randomly chosen exons was taken. A PSI value of 1 indicates constitutive exons, whereas values below 1 show exons that are not present in every transcript. Only exons of expressed genes (FPKM > = 1) were considered. If neither inclusion nor exclusion reads were present, a PSI value of 0 was assigned to indicate that the exon was not used.

### Downstream genomic variant analysis

#### Cross-species comparison

Next to the rat data described in this paper, we used variomes of dog (assembly canFam3), horse (equCab2), mouse (NCBIM37/Mm9) and pig (susScr3/Sscrofa10.2). Corresponding variants and genome sequences were downloaded from Ensembl database (release 75, ftp://ftp.ensembl.org/pub/). Variants from each of these species were transposed to mouse genome NCBIM38/Mm10 using corresponding UCSC Chain alignments. Number of polymorphic positions was calculated for sliding windows (containing 100 kb syntenic sequence, 25 Kb step between starting position of adjacent windows). Z-score transformed values were used for plotting the regions where: 1) all species showed low level of variation, i.e. were all in lower 10 percentiles. 2) all species showed high level of variation, i.e. were all in upper 10 percentiles.

#### fastStructure

We used all homozygous variants as input in the fastStructure algorithm [24] (http://pritchardlab.stanford.edu/structure.html). We determined the population structure for K = 2 until K = 31 and determined the appropriate number of model components that explain structure in the dataset by running the build-in script chooseK.py. In order to determine the genomic distributions of these clusters we divided the genome in segments containing 20.000 SNVs, in each window the genotypes of the different rat strains were compared to the average genotype profile of each of the 9 groups. Similarity scores were calculated using Spearman correlation; each window was assigned a group membership based on the maximum correlation coefficient.

#### phastCons

Conservation scores for alignments of 8 vertebrate genomes with Rat (PhastCons9way scores [34], rn4 assembly (Nov. 2004)) were downloaded from UCSC Genome browser FTP server. Since no phastCons scores were available yet for the RGSC5.0 assembly, UCSC LiftOver was used to retrieve the new coordinates of phastCons scores.

### Availability of supporting data

The genome sequence data for the four rat strains (BN-Lx/CubPrin, SHR/OlaIpcvPrin, SHR/NCrlPrin, and SUO_F344), supporting the results of this article, is available in the European Bioinformatics Institute (EBI) Short Read Archive (SRA) under accession [EBI-SRA: PRJEB6956]. The BN-*Lx*/Cub RNA-seq data, supporting the results of this article, is available in the European Bioinformatics Institute (EBI) Short Read Archive (SRA) under accession [EBI-SRA: PRJEB6938]. SNVs, indels and structural variants in all 40 strains are available by browsing via the Rat Genome Database (http://rgd.mcw.edu/) or via a direct download of the VCF file per variant type: ftp://ftp.rgd.mcw.edu/pub/strain_specific_variants/Hermsen_et_al_40Genomes_Variants/. In addition data from the four newly sequenced strains is also available via PhenoGen Informatics (http://phenogen.ucdenver.edu).

List of files which will be available through RGD (all in VCF format):

1. Homozygous SNVs annotated
2. Heterozygous SNVs annotated
3. indels
4. SVs by DELLY
5. SVs by CNVnator
6. High impact SNVs annotated
7. Substrain SNVs annotated

## Additional files

Additional file 1:
**Table with the numbers of mapped bases and average coverage of data from four rat strains.**
Additional file 2:
**Table with false positive and false negative rates of SNVs and indels per sequencing platform.**
Additional file 3:
**Table with all high impact SNVs that (not) have a variant in their vicinity that restore the open reading frame.**
Additional file 4:
**Two tables with regions with highest or lowest SNV density across six species.**
Additional file 5:
**Table with the results of the PANTHER Overrepresentation Test on 909 genes with a Ka/Ks ratio >1.0.**
Additional file 6:
**Figure showing the subchromosomal pattern of similarity and divergence between the 40 + 1 rat strains for all autosomes.**

## Abbreviations

RGSC: Rat Genome Sequencing Consortium
SNV: Single Nucleotide Variant
SNP: Single Nucleotide Polymorphism
WGS: Whole Genome Sequence
NGS: Next-generation sequencing
GATK: Genome Analysis Toolkit
PSI: Percentage Spliced In
RP: Read-pairs
RD: Read-depth
SV: Structural variant
SUO: Strain of Unknown Origin
ROS: Reactive oxygen species
QTL: Quantitative Trait Locus
VCF: Variant Call Format
RGD: Rat Genome Database
