# Supplementary material for: Genomic landscape of rat strain and substrain variation
Source: BMC Genomics. 2015 May 6;16(1):357. doi: 10.1186/s12864-015-1594-1 (PMC4422378; doi:10.1186/s12864-015-1594-1)
Supplement: Additional file 6: — Figure showing the subchromosomal pattern of similarity and divergence between the 40 + 1 rat strains for all autosomes. [file 12864_2015_1594_MOESM6_ESM.pdf]

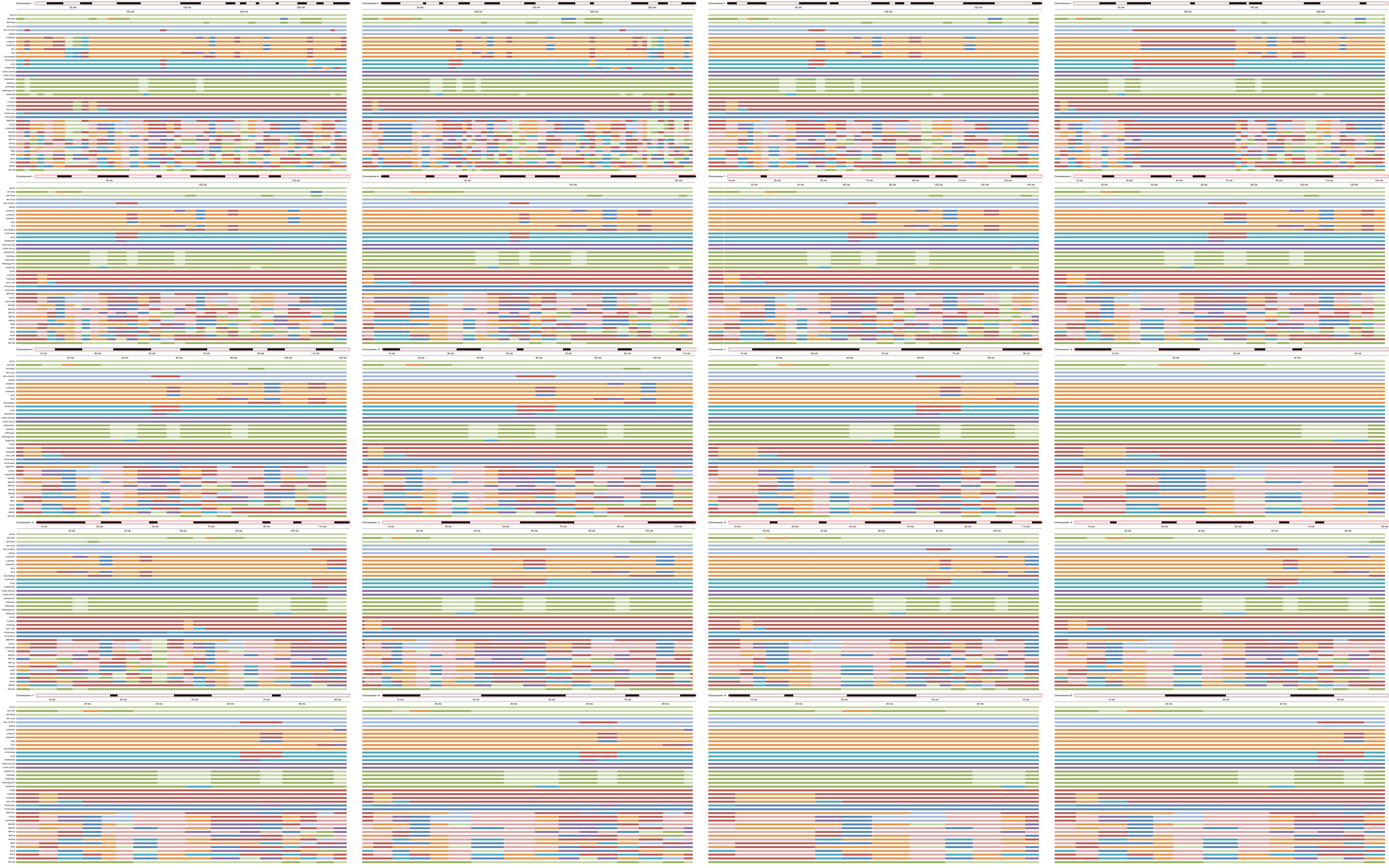
Chromosome 1
Chromosome 2
Chromosome 3
Chromosome 4
Chromosome 5
Chromosome 6
Chromosome 7
Chromosome 8
Chromosome 9
Chromosome 10
Chromosome 11
Chromosome 12
Chromosome 13
Chromosome 14
Chromosome 15
Chromosome 16
Chromosome 17
Chromosome 18
Chromosome 19
Chromosome 20
